# A genetic algorithm-based job scheduling model for big data analytics

**DOI:** 10.1186/s13638-016-0651-z

**Published:** 2016-06-27

**Authors:** Qinghua Lu, Shanshan Li, Weishan Zhang, Lei Zhang

**Affiliations:** College of Computer and Communication Engineering, China University of Petroleum, Qingdao, China

**Keywords:** Big data, Hadoop, MapReduce, Job scheduling, Genetic algorithm

## Abstract

Big data analytics (BDA) applications are a new category of software applications that process large amounts of data using scalable parallel processing infrastructure to obtain hidden value. Hadoop is the most mature open-source big data analytics framework, which implements the MapReduce programming model to process big data with MapReduce jobs. Big data analytics jobs are often continuous and not mutually separated. The existing work mainly focuses on executing jobs in sequence, which are often inefficient and consume high energy. In this paper, we propose a genetic algorithm-based job scheduling model for big data analytics applications to improve the efficiency of big data analytics. To implement the job scheduling model, we leverage an estimation module to predict the performance of clusters when executing analytics jobs. We have evaluated the proposed job scheduling model in terms of feasibility and accuracy.

## Introduction

Big data analytics (BDA) applications are a new category of software applications that process large amounts of data using scalable parallel processing infrastructure to obtain hidden value. Hadoop [1] is the most mature open-source big data analytics framework, which implements the MapReduce programming model [2] proposed by Google in 2004 to process big data. Scalability is the most important feature of Hadoop, mainly because it can easily add compute nodes in the original cluster to analyze big data.

The performance of big data analytics application is related to the characteristics of jobs and the configuration of clusters, which have a direct impact on performance of big data analytics applications. When there are multiple jobs that need to be executed with diverse cluster configurations, the solution space of job scheduling is huge and manual job scheduling is inefficient and can hardly achieve the best performance.

Genetic algorithms (GAs) [3] are used to obtain optimized solutions from a number of candidates. GAs are inspired by an evolutionary theory: weak and unit species are faced with extinction by natural selection and the strong ones have greater opportunity to pass their genes to future generations via reproduction [4]. Compared with other classic optimization methods, GAs have its specific advantages in terms of its broad applicability, ease of use, and global perspective [5]. GAs are particularly useful to one-objective and multiple-objective optimization problems [6] that make one- or multi-objective attainment to the optimum.

The contribution of this work is mainly twofold. First, we propose an estimation module to predict the performance of Hadoop clusters when executing different big data analytics jobs, which can be used by GAs. Then, with the effective information which the estimation module provides, we present a genetic algorithm-based job scheduling model for geo-distributed data.

We evaluate the proposed solution using the data centers and cluster nodes from the Amazon EC2 [7] platform. The experiment results show the proposed job scheduling model is effective and accurate.

The remainder of the paper is organized as follows: Section 2 describes the five basic stages of MapReduce data processing that can be utilized in the calculation of estimation module. Section 3 presents the genetic algorithm-based job scheduling model. Section 4 details the performance estimation module, which is used by the algorithm for the calculation of time objective. Section 5 implements and evaluates the genetic algorithm. Section 6 covers the related work. Section 7 concludes the paper.

## Related work

There have been numerous works devoted to Hadoop’s performance prediction. Berlińska and Drozdowski [8] propose a mathematical model of MapReduce and analyze MapReduce distributed computations as a divisible load scheduling problem. However, they do not consider the system constraints. There is also some work on optimizing MapReduce [9, 10]. Zaharia et al. [10] proposed a prediction model for sub-tasks of Hadoop job, rather than the entire job. Xu et al. [11] extracted characteristic values related to Hadoop performance and utilized machine learning methods to find the optimal value, without building performance models. Han et al. [12] proposed a Hadoop performance prediction model. However, it does not consider the data preparation phase of this thesis.

There are also some GA-based approaches proposed in job scheduling. Krishan Veer and Zahid [13] presents a design and eventual analysis of a scheduling strategy using GA that schedules the job with the objective of minimizing the turnaround time of the job. The evaluation is simplified due to the limitations. In [14], the application of meta-heuristic for cloud task scheduling on Hadoop is investigated. A scheduling algorithm using execution time, order of task arrival, and location of data (i.e., assign task to the node which contains the required data) to determine the best execution schedule is presented. But the performance prediction model is unintelligible, and the cost is not taken in consideration.

## Background

**Fig. 1:**
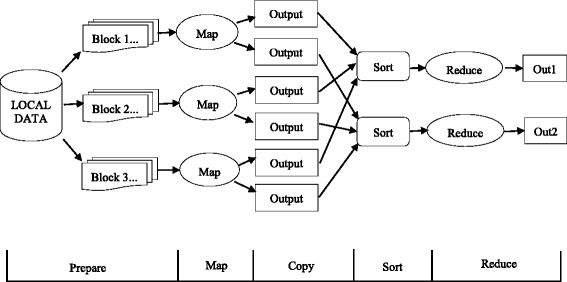
Traditional MapReduce workflow
- Map. Each mapper reads data blocks from HDFS and generates key-value pair <*k*1,*v*1> of the input data. Then, it executes a user-defined map method which generates intermediate data <*k*2,*v*2>.
- Copy. It is also called shuffle. The intermediate data from the mapper nodes is passed to the appropriate reducer based on the key. The process is from the completion of the first map wave to all intermediate data mapper outputs having been transferred to the reducer.
- Sort. This stage occurs before the reduce phase. The values of the output data from the map are sorted by the sort algorithm in accordance with the different keys and output the key-value pairs <*k*2,list(*v*2)> for the reduce phase. All the values are sorted in list(*v*2).
- Reduce. In this phase, the user-defined reduce method are executed to generate the key-value pairs <*k*3,*v*3> as the final result.

Hadoop consists of the MapReduce algorithm and the Hadoop Distributed File System (HDFS) [15]. The Hadoop workflow includes five phases, which is illustrated in Fig. 1.

- Prepare. The source data in the local disk is uploaded to HDFS in this phase. According to the predefined partition size of the data segmentation, source data are segmented in blocks first and then stored a copy to the data node in the pipeline way according to the network topology distance.

This paper utilizes the performance module for the case of this paper to predict Hadoop data processing performance including the abovementioned five phases. After the time consumption characteristics have been predicted, we can infer the cost of big data processing jobs, which depends on the characteristics of time consumption.

## Design of the genetic algorithm-based job scheduling model

### Overall design of the GA-based approach

**Fig. 2:**
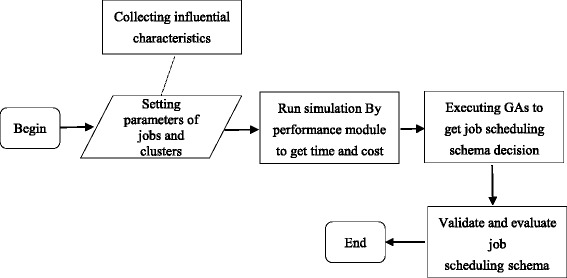
Flow diagram of the decision-making for job scheduling

The working flow of the GA-based decision-making for job scheduling is shown in Fig. 2, and the overall decision-making process is as follows. First, the estimation module is used to model the clusters and jobs. Then, some simulations are conducted to collect job execution information, such as the time and cost array which shows all the time and cost taken by each job run on all clusters. After that, the time and cost information is used in certain framework where GAs give optimized solutions for the job scheduling schema.

For big data, we choose a certain cluster deployment way to process them. However, different processing jobs do not always have the same performance with the same cluster configuration, due to the job characteristics and one job also cannot obtain the consistent result with different clusters because of the cluster or Hadoop characteristics.

When simultaneously assigning multiple jobs to process data in one data center, there are many kinds of optional cluster configuration circumstances for each job, which can bring about a large number of job scheduling schemas. So choosing the one with best performance among those jobs scheduling schema is another major issue to be addressed in this paper. In order to get optimized solutions in job scheduling decision, we use the genetic algorithm to choose solutions which have the minimal finishing time and cost. These are the objectives of the job scheduling problems.

We give a general mathematical description of the problem and then create the appropriate objective optimization model based on genetic algorithms: take the data processing in a data center, for example, and assume that a data center has *N* optional clusters and *M* jobs to be executed. We use an integer vector to represent the ultimate job scheduling scheme, *S*_*i*_ indicates that the job *i* is assigned to the cluster *S*_*i*_, where *S*_*i*_∈ [0,*M*]. So far, we have achieved getting the job scheduling scheme which have the shortest overall execution time by genetic algorithm. The overall execution time and cost refers to the time it consumed when all jobs complete the execution.

1 $$ T_{i}={\sum\nolimits}_{j=0}^{M}\text{ST}_{ij}\times E(i,j)  $$

2 $$ C_{i}={\sum\nolimits}_{j=0}^{M}\text{SC}_{ij}\times E(i,j)  $$

Here, we utilize Eqs. (18) and (19) to respectively represent the execution time and cost of all jobs assigned to one cluster, which are the objectives we considered in genetic algorithm. In order to facilitate the calculation of the overall execution time of job scheduling scheme, we add *E*(*i,j*) to indicate whether job *i* is assigned to the cluster *j*. The explanations of the symbols in the two equations are described one by one as follows:

1. *i* represents the sequence number of a job with the scope of [0,*N*].
2. *j*∈ [0,*M*] represents the sequence number of a cluster.
3. ST_*ij*_ in Eq. (18) is the time a job consumes when running in the cluster *j*. The calculation of it will be achieved by performance estimation module in the next section.
4. SC_*ij*_=*C*_*ij*_×ST_*ij*_×*N*_clus_ in Eq. (19) is to calculate the cost of running a job in the cluster *j*, in which *C*_*ij*_ represents the cost of running a certain node per second and *N*_clus_ is the number of nodes in cluster *j*.
5. When job *i* is assigned to cluster *j*, *E*(*i,j*)=1; otherwise *E*(*i,j*)=0.

A chromosome corresponds to a unique solution in the solution space. GAs can typically make use of Booleans, real numbers, and integers to encode a chromosome [9]. The representation of chromosome in our case is using integer (starting from 0) sharing the idea of [10]. That is to say, we are using an integer vector *V*=[*V*_0_,*V*_1_,*Λ*,*V*_*i*_,*Λ*,*V*_*p*−1_] (where *p* is the number of decision variables, and in our case, it is 12: the number of jobs) to represent a solution which is a natural number and acts as a pointer to the sequence of the cluster to which the job is assigned to execute. For example, a chromosome [0, 6, 2, 8, 2, 4, 5, 9, 6, 1, 3, 7] represents that a solution chooses the first cluster whose sequence number is 0 to execute job 0 and chooses the eighth cluster for job 9, the eleventh cluster as the processing cluster of the third job, and so on. Based on the chosen allocation strategies, the GAs then decide its fitness using the objective Eqs. (1) and (2) introduced.

## Performance estimation module of job execution

### Assumption

To achieve the genetic algorithm for job scheduling problem, we need to input the source data of the two objectives: time and cost array. In this paper, we propose a performance estimation module which can predict the execution time and cost of data processing jobs, according to different characteristics. The paper makes the following assumptions for simplification:

- About the reduce wave, we use recommendations in [16] and assumes that the maximum number of reduce that can be executed simultaneously is 1.
- We do not support speculative execution. That means, we will not repeat map or reduce execution and select the faster as the final result, killing the slower one, for it proved to have little contribution to improve the overall execution time.

### Total execution time overview

In this paper, performance-related parameters are divided into four categories: cluster, hadoop&HDFS, application, and obtained by module. The symbol and explanation of all parameters are listed in Table 1.

**Table 1:** Symbol and explanation of all parameters

Type	Symbol	Explanation
Cluster	DC_*i*_	the *i*th data center *i*∈[1,*N*_*dcs*_]
	*B*_*ii*_	Bandwidth between nodes in DC_*i*_
	*V*_dw_	Speed of writing data to the local disk
Hadoop&HDFS	*P*_*i*_	Partition size
	*N*_sm_	Number of simultaneous maps executed
		in one node
	*N*_cr_	Number of simultaneous reduces executed
		in one node
	$N_{\text {cp\_threads}}$	Number of i/o threads copy to one reduce
		node
	$V_{\text {cp\_thread}}$	Theoretical maximum copy speed of one
		copy thread
	$V_{\text {reduce\_rep}}$	Theoretical maximum output replication
		speed of one copy thread
	*N*_Spaths_	Number of sort paths for copy
	*N*_reps_	Number of replicas in HDFS
	*S*_buff_	Sort buffer size for copy
App	DS_*i*_	Input data size in the *i*th data center
	*N*_p_	Number of partitions
	*N*_reduces_	Number of reduces
	*M*_thruput_	Average map throughput of each node
	*R*_thruput_	Average reduce throughput of each node
	RIO_map_	Ratio of map output to input size
	RIO_reduce_	Ratio of reduce output to input size
Module	*T*_total_	Total execution time
	*T*_prepare_	Total execution time for raw data input into
		HDFS
	*T*_job_	Total execution time for a job
	*T*_map_	Time for a map wave
	*T*_copy_	Time for a copy wave
	*T*_sort_	Time for a sort phase
	*T*_reduce_	Time for a reduce phase
	*T*_rp_	Time for reduce processing
	*T*_ro_	Time for reduce output writing
	*N*_mw_	Number of map waves

The overall data processing time contains two parts: one part is the preparation time of the source data and the other is the time to perform the data processing job. Equation (3) shows the overall time to process data by clusters. The overall time for data processing in each data center needs to be calculated, and the maximum of them will be taken as the final result.

3 $$  T_{\text{total}} = {\max}(T_{\text{prepare}} + T_{\text{job}})  $$

1. Prepare time

We need to upload the data, including the replicas, from the local disk distributed in multiple data centers to their own HDFS (Eq. (4)) in this phase, where the bandwidth between node in local cluster is *B*_*ii*_.

4 $$ T_{\text{prepare}} = \frac{\text{DS}_{i} \times N_{\text{reps}}}{B_{ii}}  $$

2. Job time

(a) Map time

Map phase execution time can be calculated by Eq. (5), where the average processing time of input data block in each wave multiplied by the corresponding number of wave is the map time of the *i*th data center. The average throughput of the map is obtained from the average processing time of running the job with the input data whose block size is given. The wave number of each data center is calculated by Eq. (6) and the number of the data blocks divided is calculated by Eq. (7).

5 $$ T_{\text{map}} = \frac{P_{i}}{M_{\text{thruput}}} \times N_{\text{mw}}  $$

6 $$ N_{\text{mw}} = \left \lceil \frac{{\max}\left(N_{\text{sm}},\left \lceil \frac{N_{\mathrm{p}}}{N_{\text{nodes}}} \right \rceil \right)}{N_{\text{sm}}} \right \rceil  $$

7 $$ N_{\mathrm{p}} = \frac{\text{DS}_{i} \times N_{\text{reps}}}{P_{i}}  $$

(b) Copy time

This stage refers to the output data of the map copied to reduce. Since we only consider the case of one local cluster or different clusters in respective data center, it does not involve the transfer of data to remote and we need not consider the problem of bandwidth across the cluster between different data centers. When output data is copied to reduce, it often occurs that a plurality of thread copy data to reduce at the same time. The theoretical maximum copy speed is the sum of all thread. But in reality, copy speed is also limited by the local network bandwidth. Therefore, the actual copy speed of one thread is calculated as Eq. (8). The entire local copy speed is Eq. (8) multiplied by the number of thread (Eq. (9)). This paper argues that all nodes of a cluster are in the same subnet; thus, only the local copy speed and map output data size will have an impact on the copy time (Eq. (10)).

8 $$ \begin{aligned} V_{\text{CopyThread}} = & \,{\min}\left(\frac{B_{ii}}{{\min}\left(N_{\text{sr}},\left \lceil \frac{N_{\text{reduces}}}{N_{\text{nodes}}} \right \rceil \right) \times N_{\text{cp\_threads}}} \right.\\ & \left.,V_{\text{cp\_thread}} {\vphantom{\frac{\frac{1}{2}}{\frac{2}{1}}}}\right) \end{aligned}  $$

9 $$ V_{\text{LocalCopy}} = V_{\text{CopyThread}} \times N_{\text{reduces}} \times N_{\text{cp\_threads}}  $$

10 $$ T_{\text{copy}} = \frac{\text{DS}_{i} \times N_{\text{reps}} \times \text{RIO}_{\text{map}}}{V_{\text{LocalCopy}}}  $$

(c) Sort time

The time estimation of the sort stage is independent on the network, which is shown in Eq. (11). Among them, the calculation of the form is shown in Eq. (12).

11 $$ T_{\text{sort}}= \left\{ \begin{array}{ll} \frac{2\times \left \lceil \frac{N_{\text{reduces}}}{N_{\text{nodes}}} \right \rceil} {V_{\text{dw}}} &\times \, \lambda_{N_{\text{Spaths}}} \left(\frac{\text{DS}_{i}\times N_{\text{reps}}\times \text{RIO}_{\text{map}}}{N_{\text{reduces}}\ast S_{\text{buff}}}\right),\\ & \frac{DS_{i}}{N_{\mathrm{reduces \ast S_{\text{buff}}}}}> 1\\ & \qquad 0, \text{else} \end{array}\right.  $$

12 $$ \lambda_{F}(n,b) = \left(\frac{1}{2F(F-1)} n^{2} + \frac{3}{2}n - \frac{F^{2}}{2(F-1)}\right)\ast b  $$

(d) Reduce time

The output data of sort is the input of the reduce phase. During this phase, data processing and writing to HDFS are operated simultaneously; thus, we can take the maximum time of them and the final time of this stage (Eq. (13)).

13 $$ T_{\text{reduce}} = {\max}(T_{\text{rp}},T_{\text{ro}})  $$

Calculating the processing time of one reduce and then multiplied by the number of reduces can get the overall processing time of reduce (Eq. (14)). In this paper, it is assumed that there is only one reduce wave. The circumstances we considered is transferring all source data to a local data center to build a cluster or setting up clusters simultaneously in respective data centers without transferring source data; therefore, the source data in these two circumstances are different.

14 $$ T_{\text{rp}} = \frac{\text{DS}_{i} \times N_{\text{reps}} \times \text{RIO}_{\text{map}}}{N_{\text{reduces}} \times R_{\text{thruput}}} \times \left \lceil \frac{N_{\text{reduces}}} {N_{\text{nodes}}\times N_{\text{sr}}} \right \rceil  $$

The duplication is set as 3 in HDFS, including the original data set. In this paper, the remote cluster does not exit and all nodes are in the same subnet, so it is not time-consuming to transfer the copy to a remote cluster. One copy will be written to the local hard disk, while the remaining two copies will be stored in other nodes in the local cluster. This process should be limited to the local bandwidth. We choose the maximum time of the local disk writing time and the local clusters writing time as the reduce output writing time (Eq. (15)).

15 $$ T_{\text{ro}} = {\max} \left(T_{\text{rp\_disk}},T_{\text{rp\_local}}\right)  $$

The local disk writing time of a copy is equal to the average amount of data written to the local disk in each node divided by the disk write speed (Eq. (16)). The disk write speed is obtained by Bonnie++ [17], a tool for disk I/O performance test.

16 $$ T_{\text{rp\_disk}} = \frac{\text{DS}_{i} \times \text{RIO}_{\text{map}} \times N_{\text{reps}}}{N_{\text{nodes}}*V_{\text{dw}}}  $$

Because of the assumption that one reduce only produce a single output file and two copies of it will be copied to the other two nodes in local cluster, the copy speed is limited by the bandwidth of the subnet the cluster belongs to. We utilize Eq. (17) to estimate the minimum store speed of each reduce data copy in the local cluster.

17 $$ \begin{aligned} V_{\text{rp\_local}} = & \,{\min} \left({\vphantom{\frac{\frac{1}{2}}{\frac{2}{1}}}} V_{\text{reduce\_rep}}\right. \\ & \left.,\frac{B_{ii}}{{\min} \left(N_{\text{sr}},\left \lceil \frac{N_{\text{reudces}}}{N_{\text{nodes}}} \right \rceil \right)*(N_{\mathrm{reps-1}})} \right) \end{aligned}  $$

The reduce output data size divided by Eq. (17) is the maximum time of data copies to store in the local cluster (Eq. (18)).

18 $$ T_{\text{rp\_local}} = \frac{\text{DS}_{i} \times \mathrm{RIO_{map}}\times \mathrm{RIO_{reduce}}}{N_{\text{reduce}}}*\frac{1}{V_{\text{rp\_local}}}  $$

The copy phase from the first wave completion of the map results in the overlap between these two phases; thus, the job execution time of one cluster in respective data center is shown as Eq. (19).

19 $$ \begin{aligned} T_{\text{job}}= \left\{\begin{array}{ll} T_{\text{map}}+\frac{T_{\text{copy}}}{N_{\text{mw}}}+T_{\text{sort}}+T_{\text{reduce}}, & T_{\text{map}}>T_{\text{copy}}\\ \frac{T_{\text{map}}}{N_{\text{mw}}}+T_{\text{copy}}+T_{\text{sort}}+T_{\text{reduce}}, & T_{\text{map}}<T_{\text{copy}} \end{array}\right. \end{aligned}  $$

## Evaluation

### Setting of the experiment

Suppose we have the following hypothetical scenario: there are 12 data processing jobs and 10 clusters which have specific configuration. This is considered as one of the examples which is discussed in detail in this paper. We utilize Amazon EC2 (Amazon Elastic Compute Cloud) as a test platform. The types of the nodes in the experiment are all m1.large which is one of the node types that AWS supports and their configuration is the same to each other, namely, 64-bit RHEL (short for Red Hat Enterprise Linux) operating system, two core, 7.5-G memory, 2 420-G storages. The cost of each node is $0.34 per hour. The data center is located in US East (Northern Virginia), and the bandwidth we test using Netperf [18] is 282.4 MB/s.

The parameters of jobs and cluster configuration are listed in Tables 2 and 3, respectively. Table 4 shows other related parameters. As illustrated previously, we have 12 jobs and 10 clusters. For each job, it can be allocated to any of the 10 clusters. So, there will be allocation schemes.

**Table 2:** The parameters of jobs

Job_*i*_	DS_*i*_ (G)	RI*O*_*map*_	RI*O*_*reduce*_
0	1	0.18	0.17
1	2	0.18	0.17
2	4	0.18	0.17
3	8	0.18	0.17
4	1	1.25	1.5
5	2	1.25	1.5
6	4	1.25	1.5
7	8	1.25	1.5
8	1	1	1
9	2	1	1
10	4	1	1
11	8	1	1

**Table 3:** The parameters of clusters

Clu*s*_*i*_	*N*_nodes_	*N*_reduces_	*P*_*i*_ (M)
0	2	2	64
1	2	2	128
2	4	2	64
3	4	2	128
4	4	4	64
5	4	4	128
6	8	4	64
7	8	4	128
8	8	8	64
9	8	8	128

**Table 4:** Other related parameters

Type	Symbol	Value
Cluster	*V*_dw_	50.96MB/s
Hadoop&HDFS	*N*_sm_	4
	*N*_sr_	2
	$N_{\text {cp\_threads}}$	30
	$V_{\text {cp\_thread}}$	10 MB/s
	$V_{\text {reduce\_rep}}$	10 MB/s
	*N*_Spths_	10
	*N*_reps_	3
	*S*_buff_	716
App	*M*_thruput_	1.18 MB/s
	*R*_thruput_	15.47 MB/s

Our goal for job scheduling is to choose the best allocation scheme from all of the possible schemes using GAs. Before making this decision, we utilize the performance estimation module to predict the execution time and cost which are the decision indicators in GAs. Then, we get a two dimensional time and cost array which is shown in Tables 5 and 6, respectively. From Tables 5 and 6, we can see that different jobs running on the same cluster may not have the same time and cost consumption. Also, same jobs can take different times and costs to finish its execution in different clusters.

**Table 5:** Execution time

Time (s)	Clus_*i*_									
	0	1	2	3	4	5	6	7	8	9
Job_*i*_										
0	237.9	237.9	127.5	237.9	124.4	234.7	124.4	234.8	122.8	233.1
1	475.7	475.7	255.0	255.0	248.7	248.7	138.4	248.7	135.2	245.6
2	951.4	951.4	510.0	510.0	497.5	497.5	276.8	276.8	270.5	270.5
3	1902.8	1902.8	1020.1	1020.1	994.9	994.9	553.5	553.5	541.0	541.0
4	329.8	329.8	219.5	329.8	170.4	280.7	170.4	280.7	145.81	256.1
5	659.7	659.7	439.0	439.0	340.7	340.7	230.4	340.7	181.2	291.6
6	1319.3	1319.3	878.0	878.0	681.4	681.4	460.7	460.7	362.5	362.5
7	2638.7	2638.7	1755.9	1755.9	1362.8	1362.8	921.5	921.5	724.9	724.9
8	284.6	284.6	174.2	284.6	147.7	258.1	147.7	258.1	134.5	244.8
9	569.2	569.2	348.5	348.5	295.5	295.5	185.1	295.5	158.6	269.0
10	1138.3	1138.3	696.9	696.9	590.9	590.9	370.2	370.2	317.2	317.2
11	2276.6	2276.6	1393.9	1393.9	1181.8	1181.8	740.5	740.5	634.4	634.4

**Table 6:** Execution cost

Cost($)	Clus_*i*_									
	0	1	2	3	4	5	6	7	8	9
Job_*i*_										
0	0.04	0.04	0.05	0.09	0.05	0.09	0.09	0.18	0.09	0.18
1	0.09	0.09	0.10	0.10	0.09	0.09	0.10	0.19	0.10	0.19
2	0.18	0.18	0.19	0.19	0.19	0.19	0.21	0.21	0.20	0.20
3	0.36	0.36	0.39	0.39	0.38	0.38	0.42	0.42	0.41	0.41
4	0.06	0.06	0.08	0.12	0.06	0.11	0.13	0.21	0.11	0.19
5	0.12	0.12	0.17	0.17	0.13	0.13	0.17	0.26	0.14	0.22
6	0.25	0.25	0.33	0.33	0.26	0.26	0.35	0.35	0.27	0.27
7	0.50	0.50	0.66	0.66	0.51	0.51	0.70	0.70	0.55	0.55
8	0.05	0.05	0.07	0.11	0.06	0.10	0.11	0.20	0.10	0.18
9	0.11	0.11	0.13	0.13	0.11	0.11	0.14	0.22	0.12	0.20
10	0.22	0.22	0.26	0.26	0.22	0.22	0.28	0.28	0.24	0.24
11	0.43	0.43	0.53	0.53	0.45	0.45	0.56	0.56	0.48	0.48

In our implementation, we choose to use Java-based GA frameworks. There are some popular implementations, such as JGap [19], ECJ [20], and JMetal [21]. When compared with JMetal’s counter-parts, the design of JMetal has a good separation of concerns in terms of its easiness for applying different GAs after a problem is abstracted. Therefore, JMetal is chosen as the GA framework in our paper. In our evaluations, GA Non-dominated Sorting Genetic Algorithm (NSGA-II) is chosen as the concrete algorithm. Algorithm parameters setting in our implementation includes the maximum evaluations, the crossover probability, and the mutation operator, as shown in Table 7. As choosing integer chromosome, single-point crossover, bit-flip mutation, and Binary tournament2 operators are selected drawing on the experience of [22]. Reference [6] talks about how the operators work to make a change to the chromosome in GAs.

**Table 7:** Parameters for NSGA-II

Symbol	Value
Population size	50
Max evaluations	2000
Crossover operator	Single-point crossover
Crossover probability	0.9
Mutation operator	Bit-flip mutation
Mutation probability	1/number of variables
Selection operator	Binary tournament2

### Results

**Fig. 3:**
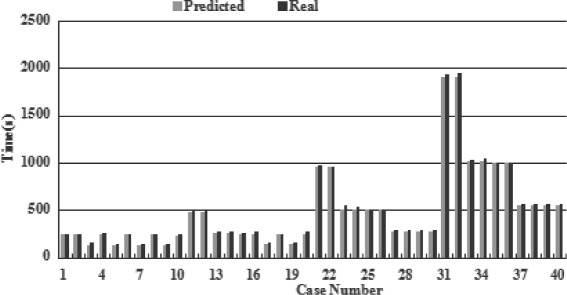
Comparison between real measurement and module prediction

In order to evaluate the effectiveness of the proposed performance estimation module, we choose US East (Northern Virginia) data center to set up real clusters in Amazon EC2 platform and run some experimental jobs and compare them to the results obtained from performance estimation module (Fig. 3). By comparing the results estimated and real results in Fig. 3, we can find that our performance module estimates accurately the data processing time in general. Bandwidth and resource load may lead to delays in real world when compared with predicted time performance, but this part of error can be acceptable. Since the estimation of cost performance depends on the times, so deduce the effectiveness of it from the above conclusion.

Meanwhile, in order to compare the performance of NSGA-II with other algorithms, we choose simple allocation method for comparison, which is a random allocation policy: all jobs are assigned to a group of clusters randomly. We verify and evaluate each job scheduling policy derived by NSGA-II and simple allocation method. The results are shown in Tables 8 and 9, respectively. From these three tables, we can find that the scheme obtained from GA-based approach takes 989.5 time units and $2.6 to finish all of 12 jobs’ execution while the other scheme from a simple method takes 2638.7 time units and $3.10. So, GA-based approach can do the optimized decision and make the data processing have the fastest execution efficiency and minimum cost. According to the GA-based scheme, data processing jobs can be finished as fast as possible with the optimized cost, and therefore, users can have better experience and can be more satisfied.

**Table 8:** NSGA-II-based approach

Job_*i*_	Clus_*i*_	Time	Cost
0	1	237.9	0.04
1	1	475.7	0.09
2	2	510.0	0.19
3	7	553.5	0.42
4	0	329.8	0.06
5	0	659.7	0.12
6	4	681.4	0.26
7	8	724.9	0.06
8	4	147.7	0.55
9	5	295.5	0.11
10	5	590.9	0.22
11	9	634.4	0.48
Total		989.5	2.6

**Table 9:** Simple scheduling method

Job_*i*_	Clus_*i*_	Time	Cost
0	1	237.9	0.04
1	9	245.6	0.19
2	2	270.5	0.20
3	7	553.5	0.42
4	7	280.7	0.21
5	7	340.7	0.26
6	9	362.5	0.27
7	1	2638.7	0.25
8	7	258.1	0.20
9	7	295.5	0.22
10	6	370.2	0.28
11	6	740.5	0.56
Total		2638.7	3.10

## Conclusions

Job scheduling is one of the most important issues in big data analytics. In this paper, we propose a genetic algorithm-based approach, which uses a performance estimation module we put forward, for obtaining optimized jobs scheduling scheme that have the optimized time and cost consumption. The optimized solutions can be used to enable effective scheduling strategies, and then in the actual running, system can make use of the chosen scheduling scheme to execute data processing jobs. The whole process is evaluated and the results show that our approach is feasible with acceptable performance and accuracy. Due to the limitations in our performance estimation module, currently, the evaluation is simplified and in the future, the approach will be extended to be more complete and precise.
